# Design and Performance Research of a New Dual-Excitation Uniform Eddy Current Probe

**DOI:** 10.3390/s22228850

**Published:** 2022-11-16

**Authors:** Tao Chen, Hai Shi, Yuanhang Dong, Cheng Lv, Zhiyang Deng, Xiaochun Song, Chunhui Liao

**Affiliations:** 1 Key Laboratory of Modern Manufacturing Quantity Engineering, School of Mechanical Engineering, Hubei University of Technology, Wuhan 430068, China; 2 Hubei Special Equipment Inspection Testing Institute, Wuhan 430077, China

**Keywords:** nondestructive testing, uniform eddy current (UEC), crack defect, direction identification, defect quantification

## Abstract

A dual-excitation uniform eddy current probe, composed of two excitation coils placed tangentially and one detection coil placed horizontally, is developed to solve the difficulties of detection rate and direction recognition of crack defect. Firstly, a probe simulation model is established using COMSOL Multiphysics, and the differences of eddy current distribution between the dual-excitation probe and the traditional probe are investigated. Then, the influence of the distance between excitation coils on sensitivity and the test capability for crack defects with different depths and directions are investigated. Besides, the sensitivity of the dual-excitation probe is compared to that of the traditional probe made of the same coils. Finally, a physical probe and an experimental system are developed, and the performance of the dual-excitation probe is tested. The experimental results show that the probe developed in this paper exhibits a slightly higher sensitivity than the traditional probe for crack defects with different depths in the range of 0.5 mm–4.0 mm; the measurement accuracy of crack length is about 3.0 mm and can avoid missing detection of crack defects with different directions. In testing, the detection signal can be compensated to achieve precision measurement by identifying the angle of crack defects. This dual-excitation uniform eddy current probe can be used for precise quantification and direction identification of crack defect in eddy current testing.

## 1. Introduction

The structural design and optimization of a probe is a key technology in the field of eddy current nondestructive testing, which determine the performance of the probe [1,2,3,4]. There are generally two common eddy current probe structures. One is the self-exciting and self-receiving single coil structure, mixing together the exciting and receiving signals, which is generally obtained by impedance analysis method [5,6]. The other is the complex structure composed of multiple coils [7,8], whose signal processing methods are more diverse and could be applied to more testing applications. The UEC (uniform eddy current) probe is a complex structure eddy current probe. Owing to the advantages of suppressing noises caused by an uneven surface of the specimen [9,10,11], various structures, and high testing efficiency, UEC probes have received extensive attention from scholars [12,13,14].

Compared with the ordinary eddy current probe whose excitation coil is generally placed horizontally, the excitation coil of UEC probe is generally placed tangentially [15,16], which changes the spatial distribution of the excitation magnetic field. The UEC probe has self-differential and self-nulling properties [17]. When the lift-off distance changes, the induced electromotive forces at both ends of detection coil cancel each other out, and the output signal is zero. In balance contagions, the output signal is also zero because of the self-nulling property, which enables the UEC probe to be immune to the test noise caused by the specimen’s uneven surface [15,18]. Reperianto et al. designed a unidirectional-butterfly double excitation UEC probe, which exhibits high detection sensitivity for aluminum plate crack defects [19]. A UEC probe based on a giant magnetoresistance sensor was developed by Postolache, O. et al. This probe can detect sub-surface crack defects of an aluminum plate using an excitation frequency below 1 kHz [20,21]. Tao, C. et al. designed an orthogonal uniform eddy current probe that can effectively detect the weld defect of a steel plate [22].

Although the traditional UEC probe can effectively suppress the test noise caused by specimen’s uneven surface, its eddy current filed is narrow and the density is small for an excitation coil that is placed tangentially. At the same time, because its test signal is affected by the angle between the scanning path and crack defect, it only shows good test capability for crack defects in some specific directions. In order to improve the performance of the traditional UEC probe, a dual-excitation UEC probe is designed in this paper. The probe consists of two excitation coils placed tangentially and one detection coil placed horizontally. When the coils are loaded with sinusoidal currents of the same amplitude and anti-phase synchronously, the crack defects in different directions present different signal waveforms. Moreover, no crack defect will be missed for the composite eddy current field under a detection coil that is annular. The research results of this paper can be used for precise quantification and direction identification of crack defects in eddy current testing.

## 2. Theoretical Research

### 2.1. The Crack Defect Test Principle of a UEC Probe

**Figure 1:**
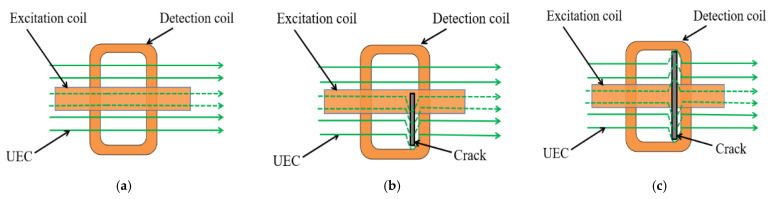
The test schematic diagram of the traditional UEC probe: (**a**) no crack defect; (**b**) asymmetric crack defect; (**c**) symmetrical crack defect.

The traditional UEC probe is generally composed of an excitation coil placed vertically and a detection coil placed horizontally or vertically, with its eddy current field parallel to the surface of test specimen. Figure 1 is the test schematic diagram of the probe with the detection coil placed horizontally. When there is no crack defect, the eddy current filed distributes symmetrically under the detection coil, and the output signal is zero for the self-nulling property [17], as shown in Figure 1a. As can been seen from Figure 1b, when there is an asymmetrical crack defect under the probe, the eddy current filed is disturbed, and the output signal changes. As shown in Figure 1c, when there is a symmetrical crack defect under the probe, although the eddy current filed is disturbed, it still shows symmetry, and the output signal is still close to zero for the self-differential property [17], which is prone to missing crack defects.

**Figure 2:**
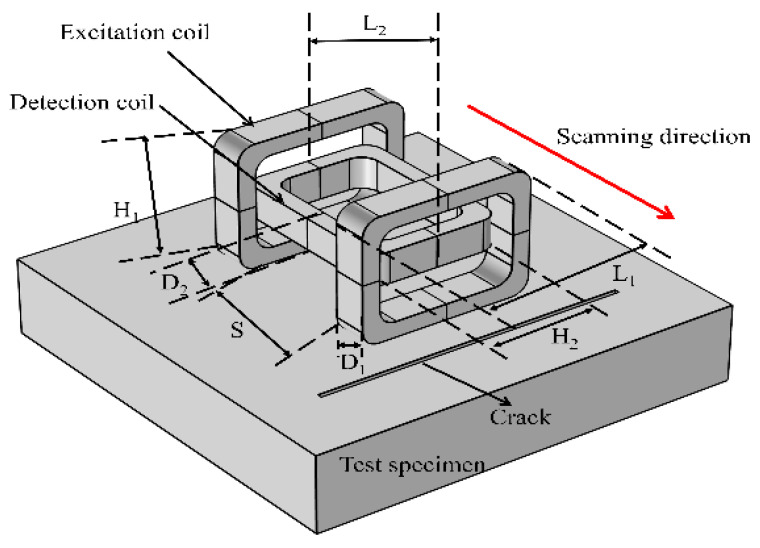
The structure diagram of the dual-excitation UEC probe.

In order to identify direction and avoid missing the detection of crack defects, a dual-excitation UEC probe shown in Figure 2 is designed in this paper. As shown in Figure 2, the probe is composed of two excitation coils placed vertically and a detection coil placed horizontally; the distance between the two excitation coils is S and the other structural parameters are shown in Table 1.

**Figure 3:**
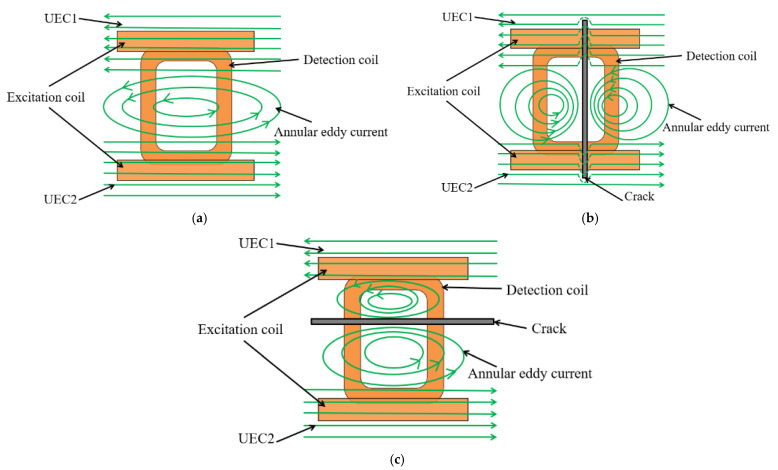
The detection schematic diagrams of the dual-excitation UEC probe: (**a**) no crack defect; (**b**) parallel crack defect; (**c**) vertical crack defect.

As shown in Figure 3a, when the sinusoidal currents with the same amplitude and anti-phase are synchronously loaded on the excitation coils, UEC1 and UEC2 with same intensity and opposite direction will be excited under two excitation coils. In addition, because UEC1 and UEC2 are in opposite directions, an annular composite UEC field with a weaker intensity than that at both ends of the detection coil will be generated, and the crack defects in any direction will cause obvious disturbance to it. As shown in Figure 3b,c, when the probe sweeps through the crack defect, the annular composite eddy current field is divided into two regions. The characteristics of the crack defect are identified by test signal, which is affected by eddy currents in two regions. Figure 3 shows that the test signal of the dual-excitation UEC probe contains more information.

### 2.2. Establishment and Characteristic Analysis of the Simulation Model

In order to study the eddy current field characteristics of the dual-excitation UEC probe, as well as the test capability for crack defects with different depths and directions, the simulation model of a steel plate specimen with crack defects is established by COMSOL Multiphysics. As shown in Figure 2, the simulation model is composed of two excitation coils, one detection coil, a steel plate specimen with crack defects, and a hidden spherical air region. Its structural parameters and material parameters are shown in Table 1 and Table 2, respectively. For the physical field boundary condition setting, the simulation model of infinite diffusion of approximate simulation of the electromagnetic field in the infinite element domain is adopted, and the impedance boundary condition is adopted for the steel plate specimen. In addition, the magnetic field in the AC/DC module is selected as the physical field. Then, the simulation model is studied in the frequency domain.

**Figure 4:**
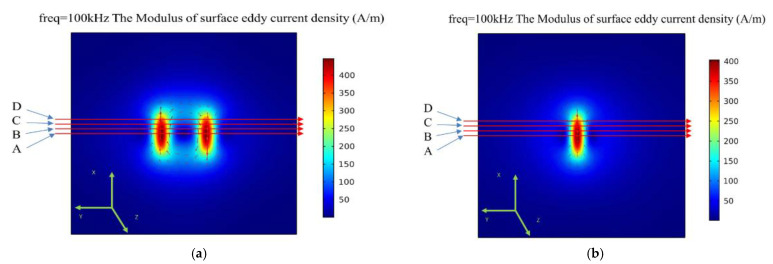
The eddy current fields of UEC probes: (**a**) dual-excitation UEC probe; (**b**) traditional UEC probe.

Firstly, the dual-excitation UEC probe is placed on the steel plate specimen without a crack defect, the lift-off distance is set to 0.5 mm, and the anti-phase sinusoidal currents with amplitude of 100 mA and frequency of 100 kHz are loaded on the probe, then the eddy current field distributed on the surface of steel plate specimen is obtained, as shown in Figure 4a. At the same time, in order to compare to the traditional UEC probe, the eddy current field of traditional UEC probe is obtained by simulation under the same conditions, as shown in Figure 4b. Comparing Figure 4a with Figure 4b, it can be seen that the eddy current field generated by the traditional UEC probe is strip-shaped, but the dual-excitation UEC probe’s eddy current field consists of two strong strip-shaped regions and a weak annular region. Compared with the traditional UEC probe, the eddy current field of the dual-excitation UEC probe contains more information.

**Figure 5:**
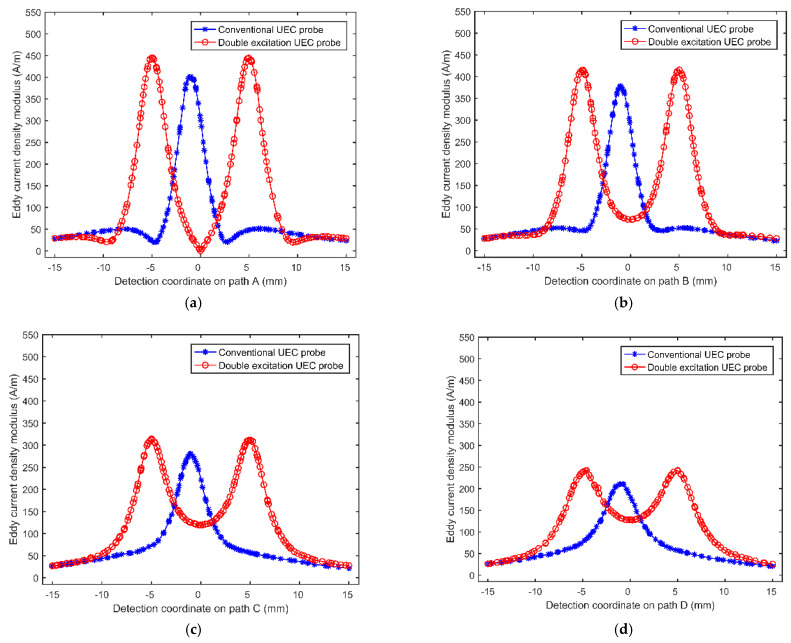
The relationship of the eddy current intensity modulus depending on the detection coordinate: (**a**) line A; (**b**) line B; (**c**) line C; (**d**) line D.

In order to further analyze the probe characteristic, the eddy current density modulus of the two probes along the four lines A, B, C, and D in Figure 4a,b are extracted using the simulation method, respectively. Line A is the symmetrical center line of the eddy current fields; lines B, C, and D are parallel to A; and the distances between lines A, B, C, and D are 2 mm. Owing to the symmetry of the eddy current fields, only the eddy current density modulus on one side of line A is analyzed in this paper. Based on simulation data, the relationship of the eddy current intensity modulus depending on the detection coordinate along the four lines of the traditional UEC probe and the dual-excitation UEC probe is obtained, as shown in Figure 5. The coordinate points 0 of the horizontal axis in Figure 5 are located on the vertical center-lines of the eddy current fields. In addition, because the two excitation coils are loaded with anti-phase sinusoidal currents, the dual-excitation UEC probe loses the self-differential and self-nulling properties. In order to facilitate a comparison to traditional UEC probe’s signals, the signal baselines of the dual-excitation UEC probe are moved to near 0 A/m in Figure 5.

It can be seen from Figure 5 that the width of the eddy current field generated by the traditional UEC probe is about 7.5 mm, and the eddy current density modulus only presents a peak near the coordinate point 0. However, the width of the eddy current field generated by the dual-excitation UEC probe is about 20.0 mm, the eddy current density modulus near the coordinate point 0 is small, and two symmetrical peaks appear on both sides of the coordinate point 0. In addition, comparing the peak values of the eddy current density modulus in Figure 5a–d, respectively, it can be found that the maximum eddy current density modulus of the dual-excitation eddy current probe is slightly greater than that of the traditional UEC probe; the reason for this phenomenon is the superposition of eddy current fields. Figure 5 shows that the dual-excitation UEC probe designed in this paper exhibits richer test information and a slightly higher test accuracy than the traditional UEC probe.

### 2.3. Optimization of Distance between Excitation Coils

The distance between the excitation coils is a key parameter that affects the performance of the dual-excitation UEC probe. The influence of the distance on the test signal is studied in this paper, and other parameters of the probe are listed in Table 1. The crack defect size of the steel plate specimen is set as length × width × depth = 20.0 mm × 0.15 mm × 1.0 mm and the excitation coils are loaded with anti-phase sinusoidal currents, of which the amplitude and frequency are 100 mA and 100 kHz, respectively. 

**Figure 6:**
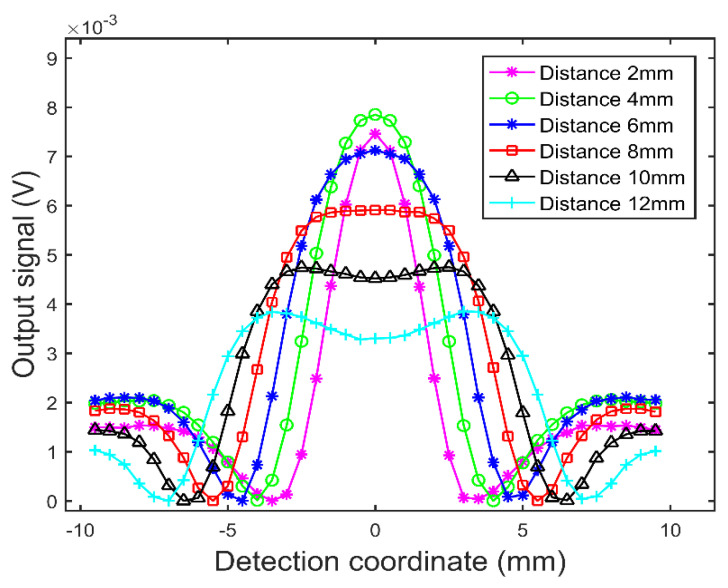
The simulation testing of vertical crack defect using the probe with different excitation coil distance.

**Figure 7:**
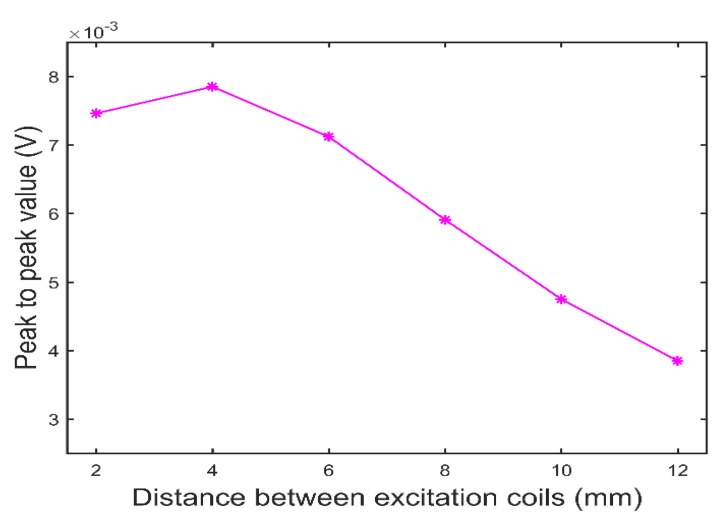
The peak to peak value of crack defect signal as a function of the distance between the excitation coils.

Then, the distance S between the excitation coils increases from 2.0 mm to 12.0 mm with a step size of 2.0 mm, and the test signals are obtained when the probe vertically sweeps through the crack defects, as shown in Figure 6. In Figure 6, the ordinate axis is the amplitude of test signal, and the abscissa axis is the probe position, where the point 0 on abscissa axis corresponds to the crack defect center. It can be seen from Figure 6 that, as the distance S increases, the amplitude of the test signal decreases, and the test signal waveform gradually widens and double peaks appear. The reason for this phenomenon is that, with the increase in distance S, the eddy current fields under the two excitation coils gradually move away from each other, resulting in the decrease in the composite eddy current field’s intensity and separation into two stronger fields. In order to determine the optimal distance S, the test signal’s peak to peak values of the probe with different distance S are extracted and the relationship between the peak to peak value of test signal and the distance S is obtained, as shown in Figure 7. It can be seen from Figure 7 that the peak to peak value of the test signal decreases with the increase in the distance S in the range of 4.0 mm–12.0 mm, but increases in the range of 2.0 mm–4.0 mm. The reason for this phenomenon is that a small distance S will cause UEC1 and UEC2 in opposite directions to cancel each other out partially, resulting in the decrease in the intensity of the composite eddy current field and the decrease in the peak to peak value of the test signal. Figure 6 and Figure 7 show that, with the increase in the distance S, the sensitivity of the dual-excitation UEC probe first increases and then decreases, and the sensitivity is highest when the distance S is 4.0 mm. However, when the distance is greater than 8.0 mm, the test signal of the probe will show an obvious bimodal phenomenon. In order to make the probe show higher sensitivity and carry richer test information, the distance S is set to 8.0 mm in this paper. The other probe parameters are shown in Table 1 and Table 2.

### 2.4. Influence of Crack Defect Depth on Test Signal

**Figure 8:**
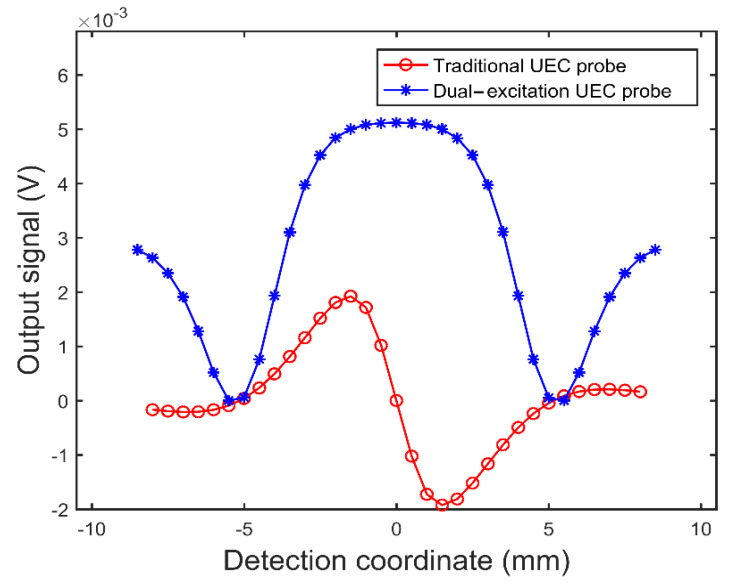
The crack defect signals of the two UEC probes.

Then, the optimized dual-excitation UEC probe is used to simulate the testing of crack defects with different depths and is compared to the traditional UEC probe made of the same coils. The crack defect lengths of the steel plate specimen are 20.0 mm; the widths are 0.15 mm; and the depths are 0.5 mm, 1.0 mm, 2.0 mm, 3.0 mm, and 4.0 mm, respectively. The two probes are loaded with sinusoidal currents, of which the amplitude and frequency are 100 mA and 100 kHz, respectively. Firstly, as shown in Figure 8, the test signals are obtained when the two probes vertically sweep through the crack defect with a depth of 0.5 mm. The coordinate point 0 of the horizontal axis in Figure 8 corresponds to the crack defect center. It can be seen from Figure 8 that the crack defect test signal of the dual-excitation UEC probe is an inverted “M” shape and the traditional UEC probe’s signal is a flat “S” shape.

**Figure 9:**
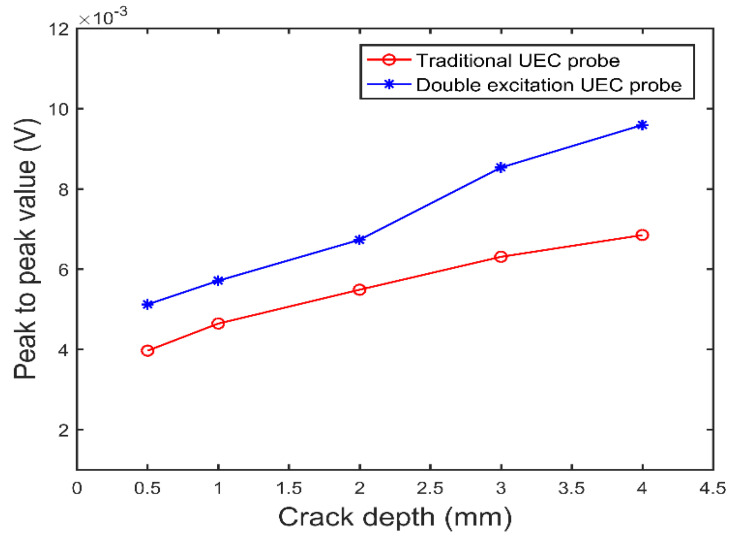
The peak to peak value of the test signal of the two UEC probes as a function of crack defect depth.

Then, the two probes vertically sweep through the crack defects with depths of 0.5 mm, 1.0 mm, 2.0 mm, 3.0 mm, and 4.0 mm, respectively. As shown in Figure 9, the relationships between the peak to peak value of the test signal and the crack defect depth are obtained. It can be seen from Figure 9 that, when the crack defect depth is in the range of 0.5 mm–4.0 mm, the peak to peak values of the two test signals increase monotonously with the increase in the crack defect depth, and the value of the dual-excitation UEC probe, which is larger for the same crack defect, increases faster when the depth is in the range of 2.0 mm–4.0 mm. Figure 9 shows that, in the crack defect depth range from 0.5 mm to 4.0 mm, the dual-excitation UEC probe designed in this paper shows higher sensitivity and better test capability for deeper crack defects.

### 2.5. Influence of Crack Defect Direction on Test Signal

Because the eddy current field of the traditional UEC probe is excited by a single coil, the eddy current field distributes uniformly under the excitation coil and parallel to it, which results in the sensitivity of the traditional UEC probe being affected by the angle between the scanning path and crack defect, and only shows good test capability in some specific directions. However, the eddy current field of the dual-excitation UEC probe shows a wider distribution area and carries more information. Then, the influence of crack defect direction on the test signal of the dual-excitation UEC probe is studied by the simulation method. The length × width × depth of crack defect is set to 20.0 mm × 0.15 mm × 1.0 mm.

**Figure 10:**
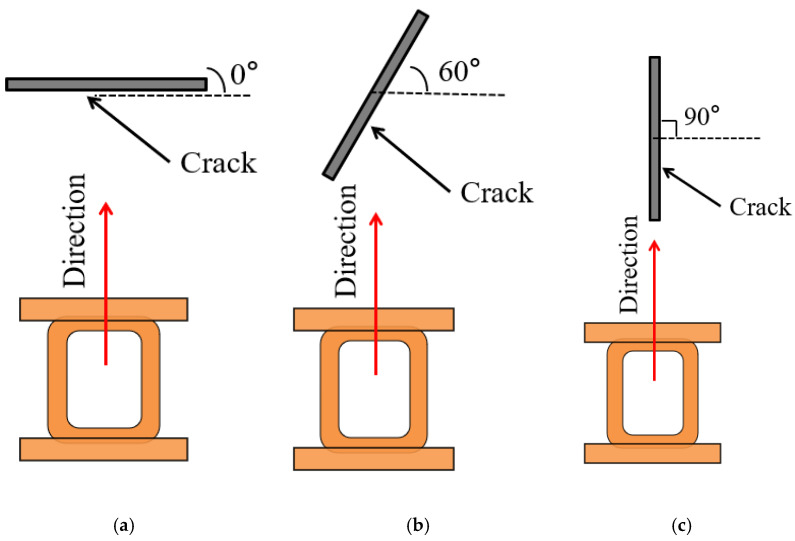
The schematic diagram of the crack defect scanning angle: (**a**) 0°; (**b**) 45°; (**c**) 90°.

**Figure 11:**
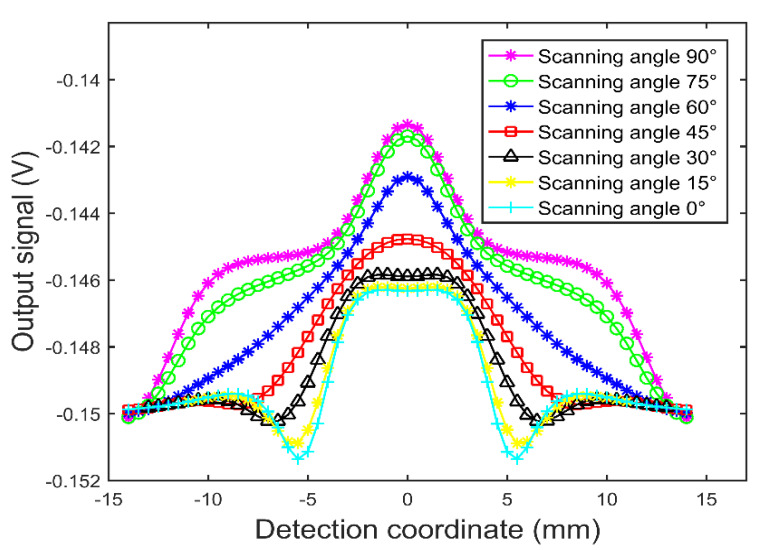
The simulated crack defect signals of the dual-excitation UEC probe at different scanning angles.

When the probe vertically sweeps through the crack defect, the scanning angle is recorded as 0° and the scanning angle increases as the crack defect rotates counterclockwise, as shown in Figure 10. Then, the dual-excitation UEC probe sweeps through the crack defects with different angles and the test signals of the scanning angle range from 0° to 90° with a step size of 15° are obtained, as shown in Figure 11. It can be seen from Figure 11 that, when the scanning angle is less than 30°, the amplitude of the test signal is small, the peak is wide, and two troughs appear on the edge of crack defect signal. When the scanning angle is in the range of 30°–60°, the amplitude of the test signal increases as the scanning angle increases, the peak narrows, and the two troughs on the edge of the crack defect signal disappear. In the scanning angle range from 60° to 90°, the amplitude of the test signal continually increases as the scanning angle increases, and the waist of the test signal extends to both ends, showing an obvious “ladder” feature. Figure 11 shows that crack defects in any direction will not be missed using the dual-excitation UEC probe designed in this paper, and the test signal waveforms show different characteristics within different scanning angles.

## 3. Experiment and Results

**Figure 12:**
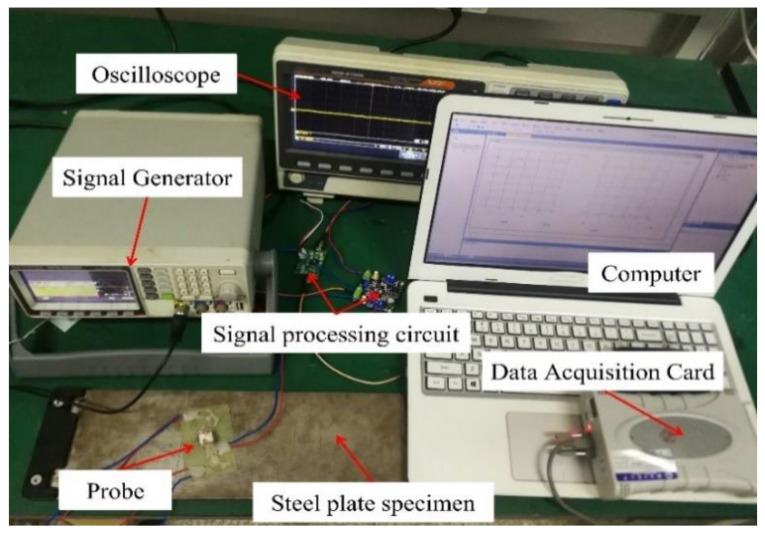
The experimental system.

In order to verify the correctness of the theoretical results, a dual-excitation UEC probe is developed according to the parameters in Table 1 and a steel plate specimen with five artificial crack defects is processed. The lengths of the crack defects are 30.0 mm; the widths are 0.15 mm; and the depths are 0.5 mm, 1.0 mm, 2.0 mm, 3.0 mm, and 4.0 mm, respectively. In addition, an experimental system that includes a signal generator, a dual-excitation UEC probe, two signal processing circuits, a data acquisition card, and a software system for the signal display is prepared, as shown in Figure 12.

### 3.1. The Testing of Crack Defects with Different Depths

**Figure 13:**
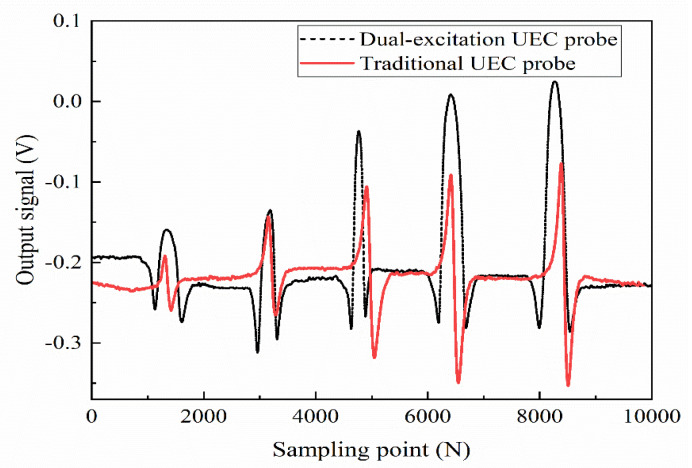
Test signals of the two UEC probes for crack defects with different depths.

Firstly, the dual-excitation UEC probe designed in this paper is used to detect the artificial crack defects with different depths. The lift-off distance of the probe is 0.5 mm, the scanning angle is 30°, and the sinusoidal currents with amplitude of 100 mA and frequency of 100 kHz are loaded on the probe. At the same time, the test results are compared to the traditional UEC probe made of the same coils, as shown in Figure 13. In Figure 13, the crack defect depths corresponding to the two signals from left to right are 0.5 mm, 1.0 mm, 2.0 mm, 3.0 mm, and 4.0 mm, respectively. It can be seen from Figure 13 that the artificial crack defects on the steel plate specimen can be effectively detected by the two UEC probes, but the signal waveforms are different. Moreover, for the same crack defect, the test signal intensity of the dual-excitation UEC probe is greater than that of the traditional UEC probe. Figure 13 shows that the dual-excitation UEC probe designed in this paper exhibits higher sensitivity than the traditional UEC probe.

### 3.2. The Testing of Crack Defects with Different Directions

**Figure 14:**
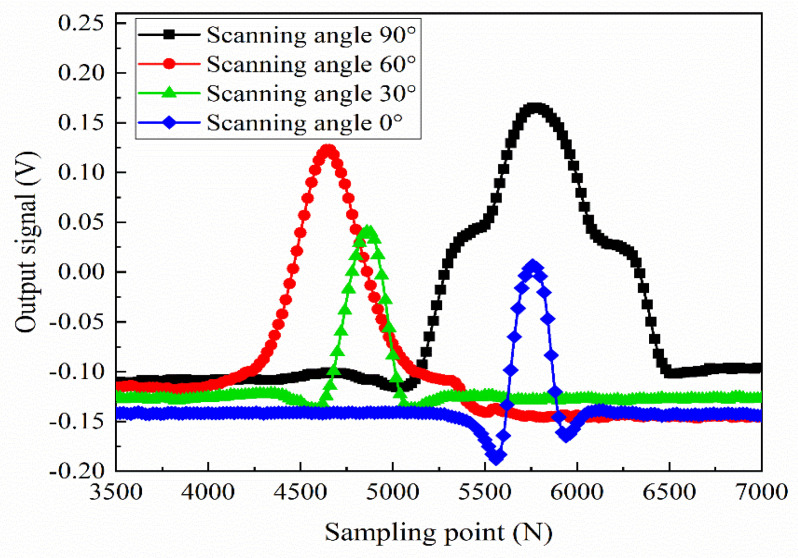
The crack defect signals of the dual-excitation UEC probe at different scanning angles.

Then, in the same test conditions, crack defects with length × width × depth of 30.0 mm × 0.15 mm × 1.0 mm are tested at scanning angles of 0°, 30°, 60°, and 90°, respectively. As shown in Figure 14, the test signals at different scanning angles are obtained. It can be seen from Figure 14 that, when the scanning angle is 0°, the test signal intensity is the smallest, but the crack defect can be effectively identified, and the reverse double peaks feature is obvious. When the scanning angle is 30°, the test signal intensity increases slightly and still shows a reverse double peaks feature. When the scanning angle is 60°, the reverse double peaks disappear, the test signal only shows a positive single peak feature, and the test signal intensity increases significantly. When the scanning angle is 90°, the waist of the single peak signal expands outward, showing an obvious “ladder” feature. Comparing Figure 14 to Figure 11, it can be seen that the experimental results are in good agreement with the simulation results. Figure 11 and Figure 14 show that the dual-excitation UEC probe designed in this paper can effectively identify the crack defect direction within a certain angle range, and there will be no missing crack defect. Besides, the probe shows the highest detection sensitivity when the scanning angle is 90°.

### 3.3. The Testing of the Probe’s Measurement Accuracy

**Figure 15:**
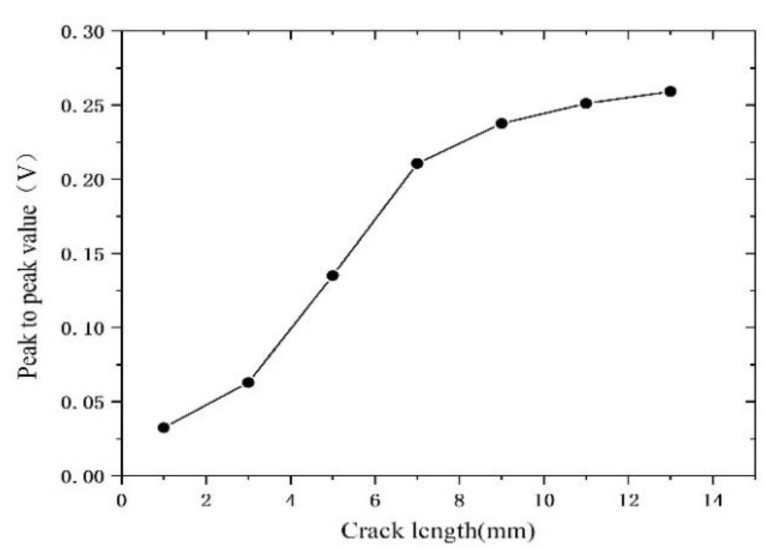
The peak to peak value of the probe’s test signal as a function of the crack length.

In order to determine the measurement accuracy of the probe, in the same test conditions, the crack defects, with widths of 0.15 mm; depths of 1.0 mm; and lengths of 1.0 mm, 3.0 mm, 5.0 mm, 7.0 mm, 9.0 mm, 1.1 mm, and 1.3 mm, respectively, are tested at a scanning angle of 90°, through which the relationship between the peak to peak value of the test signal and the length of the crack defect is obtained, as shown in Figure 15. It can be seen from Figure 15 that, when the crack length is in the range of 3.0 mm–8.0 mm, the peak to peak value of the test signal rapidly increases as the crack length increases. When the crack length is in the range of 8.0 mm–13.0 mm, the peak to peak value also increases as the crack length increases, but the increasing speed is obviously slower. Further, when the crack length is less than 3.0 mm, the peak to peak value attenuates to about 20%, the signal-to-noise ratio of the probe is poor, and it is difficult to effectively identify crack defects. Figure 15 shows that the measurement accuracy of the dual-excitation UEC probe designed in this paper is about 3.0 mm.

## 4. Discussion

The UEC probes show good accuracy and capability of noise suppression for self-differential and self-nulling properties, and they are widely used for testing specimens with an uneven surface, such as welds. However, because the eddy current field of the traditional UEC probe is excited by a single coil, it distributes uniformly under the excitation coil and parallel to it, which results in the traditional UEC probe only showing good test capability in some specific directions and being prone to missing crack defects. In order to solve the difficulties of the detection rate and direction recognition of crack defect in eddy current testing, a dual-excitation UEC probe that loses the self-differential and self-nulling properties is designed in this paper. An annular composite eddy current field is excited under the detection coil for the structure of double excitation coils, and the crack defects in any direction will cause an obvious disturbance to the field. At the same time, their test signals will show different waveform characteristics, and the crack defect’s angle can be determined according to these characteristics. In testing, the detection signal can be compensated to achieve high precision measurement [23] by identifying the angle of crack defects.

## 5. Conclusions

A new type of dual-excitation UEC probe is designed in this paper. The probe shows the advantages of high detection accuracy, no missing crack defect, and the ability to identify crack defect direction. Firstly, the crack defect test principle and the eddy current field distribution characteristics of the dual-excitation UEC probe are investigated by the simulation method. Then, compared with the traditional UEC probe, it is found that the probe with a dual-excitation coil structure shows better test capability and carries richer information. Finally, a physical probe and a test system are developed to verify the rationality of the simulation results, and experimental studies are carried out. The experimental results show that the probe developed in this paper exhibits a slightly higher sensitivity than the traditional probe for crack defects with different depths in the range of 0.5 mm–4.0 mm, the measurement accuracy of crack length is about 3.0 mm, and it can avoid missing the detection of crack defects with different directions. In testing, the detection signal can be compensated to achieve precision measurement by identifying the angle of crack defects. This dual-excitation uniform eddy current probe can be used for precise quantification and direction identification of crack defects in eddy current testing.

## Figures and Tables

**Table 1:** The structural parameters of the simulation model.

Parameter	Excitation Coil	Detection Coil
Length (mm)	L1 = 10	L2 = 10
Width (mm)	D1 = 2	D2 = 2
Height (mm)	H1 = 8	H2 = 7.6
Air area radius (mm)	R = 50	
Spacing (mm)	S = 8	

**Table 2:** The material parameters of the simulation model.

Material	Attributes	Conductivity (S/m)	Relative Permeability
Coil	copper	5.998 × 10^7^	1
Carbon steel plate	iron	1.12 × 10^7^	500
Air area	air	10	1
